# A High Performance Triboelectric Nanogenerator Using Porous Polyimide Aerogel Film

**DOI:** 10.1038/s41598-018-38121-1

**Published:** 2019-02-04

**Authors:** Zia Saadatnia, Shahriar Ghaffari Mosanenzadeh, Ebrahim Esmailzadeh, Hani E. Naguib

**Affiliations:** 1 Department of Mechanical and Industrial Engineering, University of Toronto, 5 King’s College Road, Toronto, ON M5S 3G8 Canada; 2 Faculty of Engineering and Applied Science, University of Ontario Institute of Technology, 2000 Simcoe St. N. Oshawa, Ontario, L1H 7K4 Canada

## Abstract

This paper presents a novel aerogel-based Triboelectric Nanogenerator (TENG) which shows a superior performance for energy harvesting and sensing applications. Polyimide-based aerogel film with varying open-cell content level is developed to be used as the main contact material for the TENG. The fabricated aerogel film is fully characterized to reveal the chemical and mechanical properties of the developed material. It is shown the use of Polyimide aerogel film remarkably enhances the performance of the TENG compared to a TENG with fully dense Polyimide layer with no porosity. This enhancement is due to the increase on the effective surface area, charge generation inside the open-cells of the aerogel, and increase on the relative capacitance of the TENG device. The effect of varying porosity from zero to 70% of open-cell content reveals that the aerogel film with 50% shows the highest performance where the peak open-circuit voltage of 40*V* and peak short-circuit current of 5 *μA* are obtained. These values are higher than those of the TENG with simple Polyimide layer with an order of magnitude. Finally, the performance of proposed TENG under resistive loads and capacitors are tested. Thus, this work presents an effective method for high performance TENG.

## Introduction

With the negative impact and limitation of fossil fuel-based energy sources and batteries, it has always been desired to propose environmentally friendly and renewable energy sources for macro and micro scale applications. Therefore, design and development of energy harvesting systems for scavenging energy from available clean energy sources is a very attractive research topic for the researchers. Within all clean energy sources, the mechanical energy is the most available and widely distributed energy around the world including human activities, automotive vehicles, industrial machines, structural vibrations, water wave, and wind motions^1^. Therefore, various researches have been dedicated to propose efficient energy harvesting technologies or to enhance the performance of current systems^2^. These researches are mostly based on the conventional electromagnetic or piezoelectric energy conversion technologies and have been used for different sources to some extent^3,4^.

Recently, Triboelectric Nanogenerator (TENG) has been introduced as a novel and efficient method for converting mechanical energy into electricity^5^. TENG is based on the combination of contact electrification and electrostatic induction and has shown a great potential for various energy harvesting and sensing systems^6,7^. The high efficiency of TENG has resulted in the use of this technology in a variety of applications such as energy harvesting from human activities^8^, ocean waves^9^, wind motion^10^, and vibration^11^. Also, the accuracy and precision of TENG-based sensors have led to the development of various sensory systems for measuring pressure and force^12^, trajectory and speed^13^, chemical parameters^14^, and so on^15^. In addition, TENG has shown a promising potential for being combined with other energy harvesting and sensing technologies such as electromagnetic and piezoelectric techniques^16^. Thus, different platforms have been proposed recently namely hybridized triboelectric-electromagnetic or triboelectric-piezoelectric energy conversion systems for a number of applications^17,18^.

Due to the importance and capabilities of TENG for various devices, proposing novel methods to improve the performance of TENG-based systems has always been indispensable and different directions have been considered to fulfil this target. TENG is essentially based on the contact- separation or relative sliding between two distinct materials. Therefore, advanced structural design has been utilized to propose further optimized devices which can effectively transfer the mechanical kinetic energy into the device^9^. For example, interdigitated structures^19^, zigzag shape devices^20^, rolling element configurations^21^, spring-assisted mechanisms^22^, and other advanced structures^23^, have been proposed for better energy transfer and conversion in TENG systems.

In addition, TENG technology is based on an interfacial phenomenon i.e. triboelectrification and therefore, the surface properties of interacted materials play a very vital role in the out performance of TENGs^24^. Accordingly, different methods have been developed to improve the surface properties of contact materials, those are mainly based on physical surface modification or chemical surface modification^25,26^. For instance, furnishing the contact surfaces of materials with micro/nano structures can physically boost the TENG performance owing to increase on effective contact area during the interaction of materials under mechanical stimuli^27^. Also, adding functional groups with excellent electron donation/attraction properties into the surface of materials can chemically enhance the TENG outputs due to extensive charge generation on the surfaces after being in contact with other materials^28^.

Moreover, development of advanced and smart materials, which show high performance while being used in the TENG devices, is a very useful method for enhancing the output performance of the TENG^29^. Such advanced materials can positively influence different parameters in a TENG device such as relative capacitance, dielectric constants, and surface morphologies of the TENG layers due to their exclusive properties and compositions^26,30,31^. As examples, shape memory polymers have been employed recently to effectively harvest the mechanical energy based on the TENG operation^32^. Fiber-based materials were introduced into the TENG as the main contact material to upgrade the outputs of the TENG^33,34^. Foam-shaped materials with nano/micro-porous spongy configurations were also presented as effective materials which can be used as the contact layers of the TENG device^35–37^. Such spongy structures were mostly fabricated by mixing PDMS with micro/nano particles such as NaCl microparticles and ZnO nanoparticles to be embedded first and then removed from the PDMS structure^35,38^. For example, using ZnO nanoparticles for fabricating spongy PDMS resulted in the electrical output 3.7-fold of the TENG output with a flat PDMS film^38^. Applying polystyrene micro/nano spheres for porous PDMS fabrication led to the electrical output 5 times as much as that of the solid PDMS film TENG^39^. A spongy PTFE film in a single-electrode TENG achieved the output voltage 1.8 times more than that of the TENG with simple PTFE film^40^. Also, embedding nanoparticles of silver (Ag), SrTiO_3_ and gold (Au) into spongy PDMS enhanced the electrical outputs of the TENG for 4, 5, and 5 times, respectively^29,35,41^. In addition, a TENG using PDMS composite film with graphite particles reached the output 2.6-fold of the pure PDMS film TENG^42^.

In this study, an advanced material based on Polyimide aerogel is developed to be utilized as the main contact material for the TENG. The Polyimide film contains nano-scale pores distributed through the material structure known as open-cell content without the need of using any external micro/nano particles. A relatively high and controlled open-cell content is obtained inside the material which provides exclusive properties to significantly enhance the output performance of the TENG. Initially, the fabrication process of the aerogel film is fully elaborated. Then, the chemical, thermal, and mechanical properties of the film are characterized. Afterward, the developed aerogel film with different open-cell content is used in the TENG and the effects of varying open-cell content on the electrical outputs are fully investigated. After determination of the optimum the open-cell content, the effects of various mechanical and electrical parameters such as frequency of operation and external resistive loads on the performance of the TENG are investigated. Finally, the ability of the aerogel-based TENG for charging capacitors are shown. Hence, the developed approach remarkably boosts the performance of the TENG which can be used for various energy harvesting and sensing applications. In fact, the proposed aerogel-based TENG demonstrates a novel material for designing high performance triboelectric nanogenerators to be employed in vibration energy harvesting, energy harvesting from human activities, and so on.

**Figure 1:**
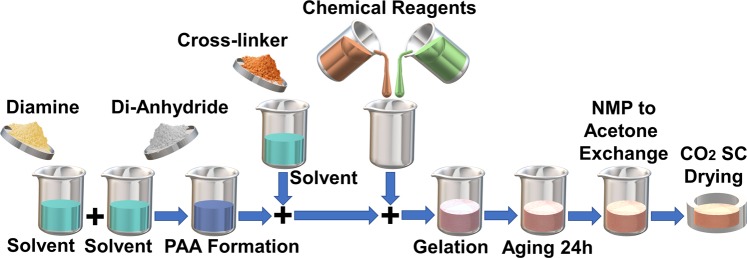
Schematic preparation process of the Polyimide aerogel film.

**Figure 2:**
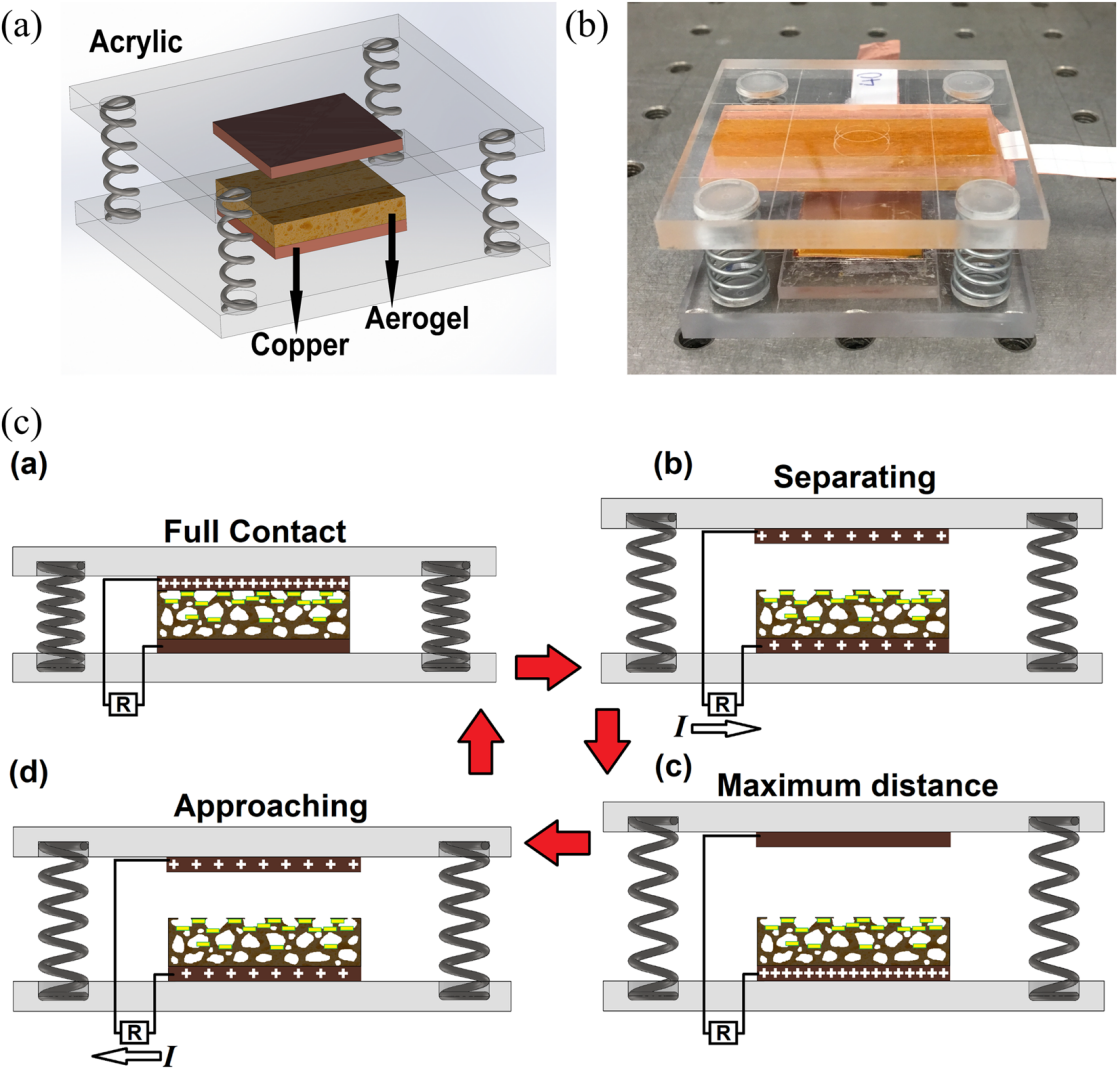
(**a**) Schematic configuration of the TENG device, (**b**) fabricated device, (**c**) working mechanism of the metal to aerogel dielectric contact-mode TENG.

The schematic preparation process of the aerogel film is depicted in Fig. 1 and the detailed fabrication process has been discussed in the methods section accordingly. In addition, the schematic components and the fabricated contact-mode TENG device are depicted in Fig. 2(a,b), respectively. The metal-to-dielectric contact-mode TENG were considered for the device. The Polyimide-based aerogel film has a negative electron affinity according to the triboelectric properties of materials. Due to high conductivity and appropriate positive electrode affinity, Copper film was used as the TENG electrode as well as the contact material with the aerogel film. The 500 *μm*-thick aerogel film was cut into 20 *mm* × 20 *mm* and a copper film with the same area was placed on the back surface of it to act as the bottom electrode. Another copper film with the same area were used to act as the contact layer and the top electrode simultaneously. Also, two Acrylic sheets were cut into 50 *mm* × 50 *mm* to be used as the top and bottom supports for the tribo-layers and electrodes. Finally, four identical compressive springs were placed at each corner of the device to provide the contact and separation under the external mechanical loads and assist the oscillatory excitation applied by a shaker for further studies shown in next sections.

The operation of the metal-to-dielectric contact-mode TENG for one cycle is shown in Fig. 2(c) where the detailed working mechanism can be found in the literature^5^. Also, the influence of nano-pores on enhancing the induced charges on the outer contact surface and porous surface of the film is schematically shown in this figure^36^. Based on the contact electrification, when the top and bottom layers are brought into contact, electrons are transferred from copper surface to aerogel surface and create triboelectric charges on the two surfaces owing to the difference between electron affinities of the two materials. As the two surfaces are separated, a potential difference occurs between the two layers which drives the electrons between the electrodes due to electrostatic induction until reaching the balanced condition. When the two layers approach each other, the electrons flow in the opposite direction until the full contact is achieved. Therefore, the repetitive contact and separation of the effective layers will result in an alternating current flow though the external circuit between the two electrodes as the generated electricity.

**Figure 3:**
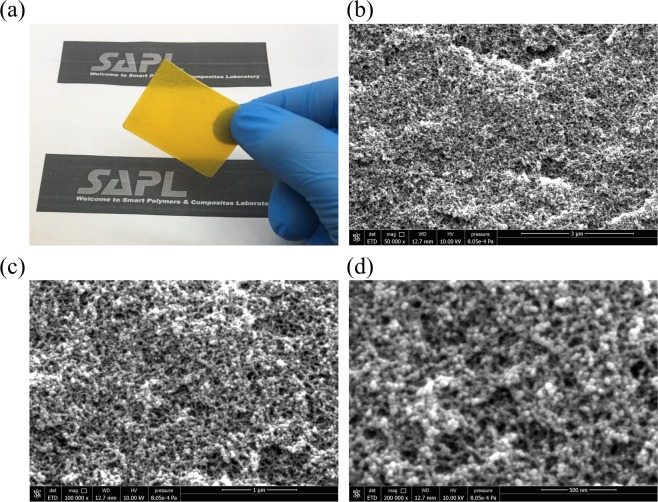
(**a**) Fabricated aerogel film sample. SEM images of the aerogel morphology for different resolutions: (**b**) 3 *μm* scale bar, (**c**) 1 *μm *scale bar, (**d**) 500 *nm* scale bar.

Figure 3(a) represents a sample of the fabricated aerogel film based on the procedure discussed in the previous sections. The measured open-cell content by helium pycnometer has shown the value of nearly 87% for the sample which proves the high level of porosity in the layer and therefore very light weight of the film. Also, Fig. 3(b) to (d) depicts the SEM images of the aerogel morphology for different resolutions in which the darker zones in the images are relevant to the air cells. As it is clear from the images, the nano pores are fully distributed through the layer and an interconnected structure is evidently observed in the images. The higher resolution shown in Fig. 3(d) can further elaborated the configuration of the aerogel structure and the distribution of nano pores inside the material It should be noted that such pores not only appear inside the structure, but also exist on the surface of the layer which can favorably increase the surface area of the contact layer while being used in the TENG device.

**Figure 4:**
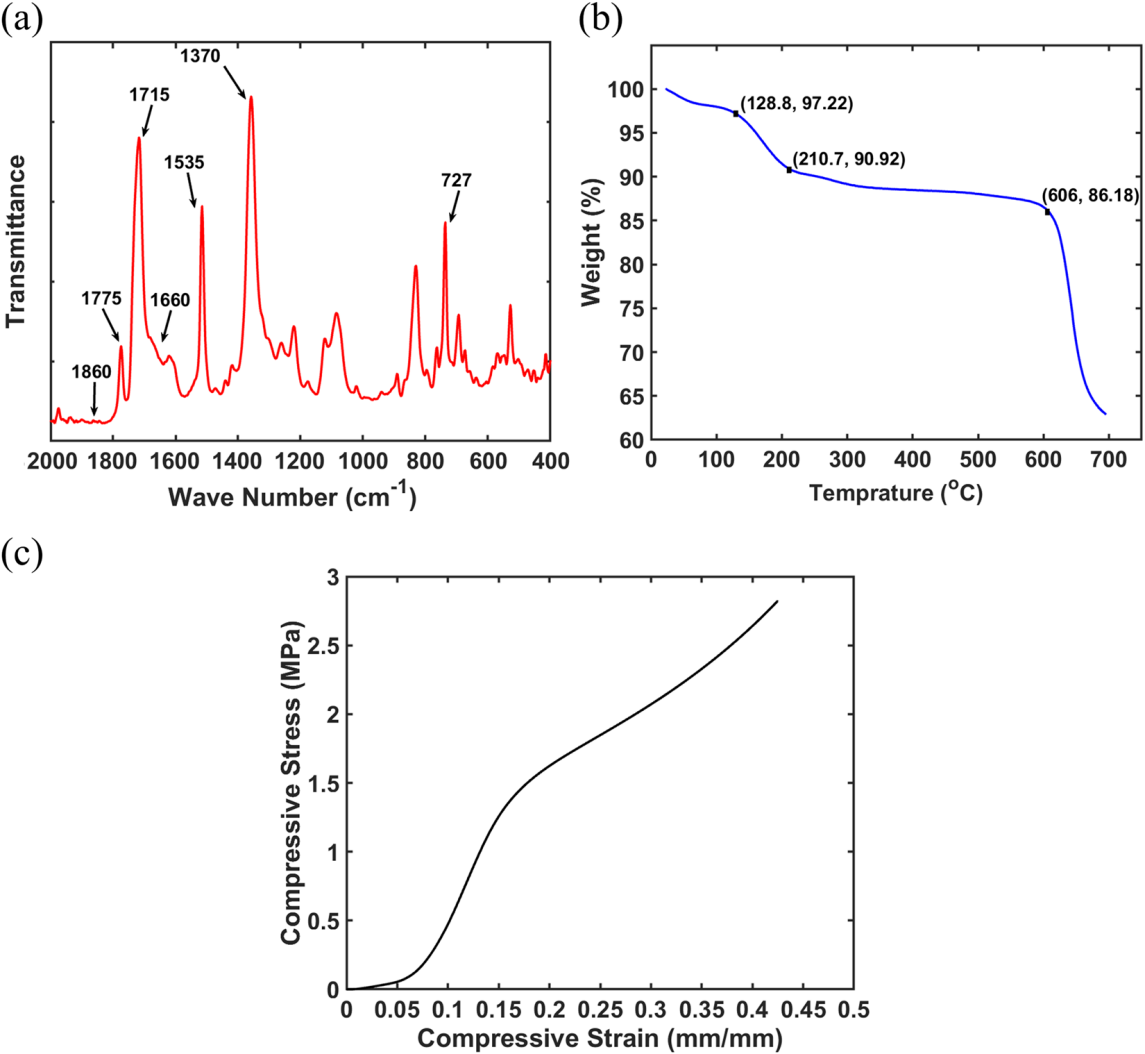
(**a**) Fourier transform infrared (FTIR) spectrum of aerogel, (**b**) TGA analysis of the sample, (**c**) mechanical compression test analysis.

Figure 4(a) shows the FTIR spectroscopy measurement of the sample to clarify the chemical structure of the fabricated aerogel. Various peaks are observed with respect to the variation of the wave number. Accordingly, the characteristic bonds of the polyimide such as 727 (cm^−1^), 1370 (cm^−1^) (imide C−N), 1715 (cm^−1^) (symmetric imide C=O) and 1775 (cm^−1^) (asymmetric imide C=O), are observed in the figure^43^. However, some picks are found around 1535 (cm^−1^) (amide C−N) and 1660 (cm^−1^) (amic acid C=O) bonds which can be attributed to the incomplete imidization of the samples. In addition, no peak is observed around 1860 (cm^−1^) indicating that no unreacted anhydride exists in the structure.

Figure 4(b) represents the TGA test results considering a heat ramp at the rate of 10 °C/min under nitrogen environment when the initial condition is the room temperature and the final temperature is 700 °C. It is evident that the onset temperature where the thermal decomposition of the sample occurs is around 606 °C which is relatively high for the sample. There is a small weight loss of nearly 7% within the range of 127 °C to 210 °C which can be attributed to either the presence of NMP or incomplete in the sample. Therefore, the thermal stability of the fabricated aerogel for a wide temperature range can be approved through this analysis.

Due to the application of proposed aerogel film for contact-mode TENG system, it is important to realize that the fabricated material is relatively strong under compression loads. Accordingly, a standard mechanical compression test was carried out on a sample of fabricated aerogel material. Figure 4(c) depicts the strain-stress curve of the compression test applied on the sample and accordingly, the average Young’s modulus of 15.87 *MPa* and average yield stress of 1.28 *MPa* were obtained for the material. These values suggest a relatively high strength of the proposed aerogel and therefore the fabricated material shows appropriate mechanical properties for further applications under external mechanical loads.

## Results and Discussion

**Figure 5:**
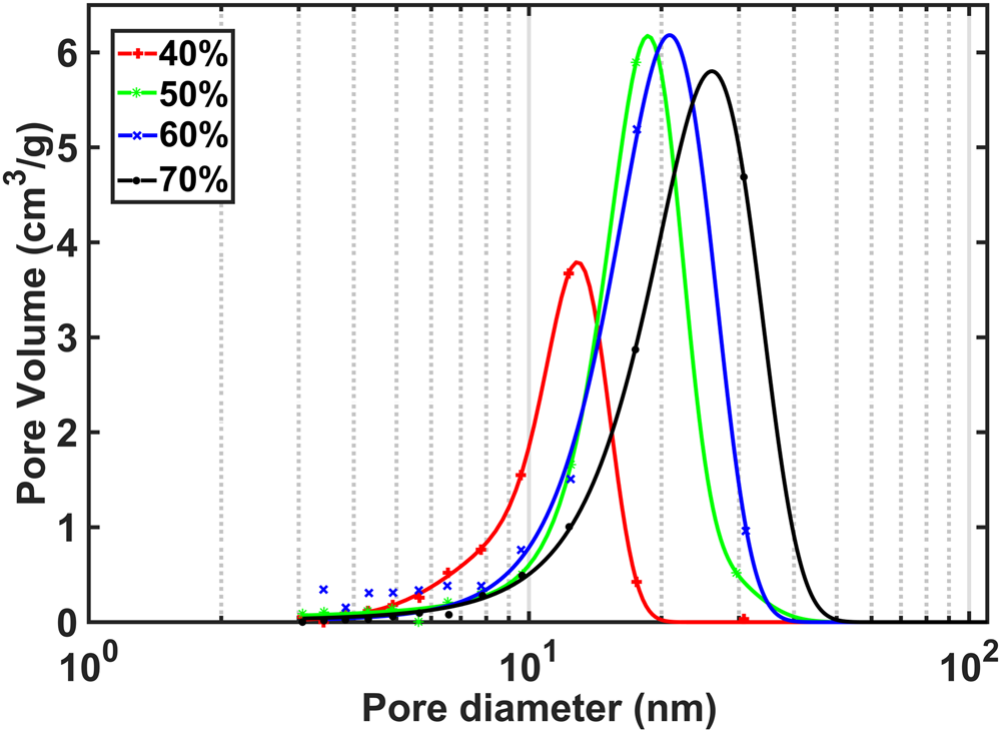
Pore size distribution for aerogel samples with different open-cell contents.

The electrical output characteristics of the aerogel-based TENG is evaluated in this section. To study the effect of porosity on the output performance, the aerogel film samples were compressed at different rates to partially remove the pores and obtain samples with different open-cell contents. The samples were then evaluated by the pycnometer and accordingly, four samples with the open-cell contents of nearly 40%, 50%, 60%, and 70% were obtained. The introduced polyimide aerogel films are mainly involved in mechanical compression according to the targeted TENG design and configuration. Therefore, mechanical compression test was performed on the introduced aerogel samples and the modulus of elasticity of each sample was obtained based on the initial slope of the stress-strain curves, as seen in Table 1. Furthermore, the surface area of the aerogel films with different open-cell contents was measured by nitrogen sorption and analyzed based on Brunauer−Emmett−Teller (BET) method^44^, and the results are also presented in Table 1. Accordingly, reducing open cell content results in increasing density and modulus. However, reducing open cell content leads to decreasing the surface area where surface area ranging from 380.5 m^2^/g to 244.5 m^2^/g is observed. Furthermore, the pore size distribution of different samples has been represented in Fig. 5 where the pore size distribution is mainly centred at about 20 nm pore diameter for the samples. It should be noted that the electrode side of compressed layers were carefully sanded and measured to achieve same thickness for all dielectric layers. For comparison, a sample was fully compressed so that the open-cell content was found less than 1% to be considered as the material with no porosity or dense material. All prepared samples were then placed into independent TENG devices and experimented separately to be compared with each other in terms of their electrical outputs.

**Table 1:** Properties of aerogel samples with different open-cell contents.

Open-cell content (%)	Density (g/cm^3^)	Young’s Modulus (MPa)	Surface area (m^2^/g)
40	0.424	28.39	244.5
50	0.409	27.4	308.7
60	0.366	24.5	362.3
70	0.323	21.65	380.5

**Figure 6:**
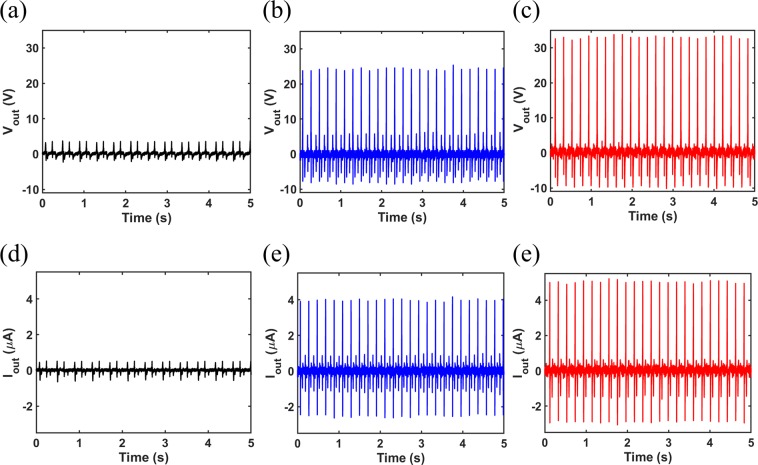
Output Voltage signals for (**a**) dense, (**b**) 40%, and (**c**) 50% open-cell contents. Output current signals for (**d**) dense, (**e**) 40%, and (**f**) 50% open-cell contents.

Figure 6(a–c) show the open-circuit voltage signals for the dense, 40%, and 50% open-cell content films, respectively. The peak voltages of 3.7*V*, 24.6*V*, and 33.8*V* are obtained for the dense, 40%, and 50% cases, respectively. Also, Fig. 6(d–f) depict the short-circuit currents of the three cases where the peak currents of 0.5 *μA*, 4 *μA*, and 5 *μA* are found for the dense, 40%, and 50% open-cell content films, respectively. It should be noted that in all experiments, the frequency and acceleration of excitation were typically selected as 5 Hz and 0.5 g, respectively. It is clear that using the aerogel film can significantly enhance the TENG performance, and this improvement it directly related to the percentage of porosities. In fact, the peak output voltage and peak output current have increase with almost an order of magnitude when the open-cell content of the aerogel is 50%. Therefore, using the proposed aerogel film can remarkably boost the electrical outputs of a TENG compared to the simple material with no porosities. Such increase on the outputs by using the proposed aerogel can be attributed to different reasons. First, the existence of pores on the surface of the contact layer can increase the contact area which leads to further charge generation on the bottom surface as a result of triboelectricity. Also, the compression of inner pores of the aerogel film while being under the contact by the top layer can result in charge generation on the surfaces of inner open-cells which enhances the electrical potential difference between the aerogel film electrode and the top copper layer owing to the electrostatic effect^36^. In addition, the existence of air cells reduces the effective thickness of the bottom dielectric which will improve the relative capacitance of the whole TENG under operation, and therefore the output of the device will be improved significantly in comparison with a simple dense layer with a compact structure^35^.

**Figure 7:**
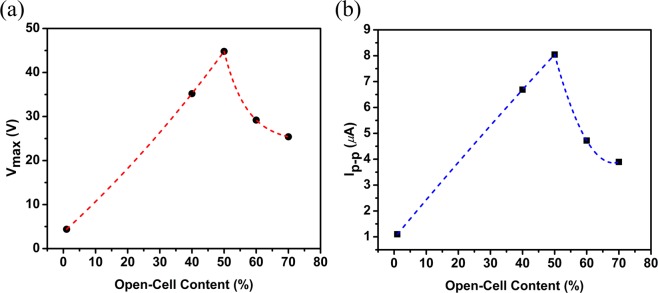
Effect of open-cell content percentage on: (**a**) peak-peak output voltage, (**b**) peak-peak output current of the TENG.

To further evaluate the effect of open-cell content on the electrical outputs of the TENG, two more samples with higher open-cell contents including 60% and 70% were examined. The results of the experiments on all sample are summarized in Fig. 6 where Fig. 7(a) shows the variation of peak-peak open-circuit voltage signal with respect to the open-cell content percentage, and Fig. 7(b) depicts the variation of peak-peak short-circuit current signal versus the open-cell content. As the open-cell content changes from zero to 50%, both peak-peak voltage and current are improved significantly. At 50%, the peak-peak voltage and peak-peak current are nearly 45*V* and 8 *μA*, respectively. After that, the electrical outputs decrease remarkably by increasing the open-cell content. This trend can be attributed to the variation of the capacitance properties of the TENG device. Indeed, one of the main techniques for enhancing the TENG performance is to improve the intrinsic capacitance of the TENG system^35^. This parameter is directly related to the effective dielectric constant or relative permittivity of the applied dielectric layer, and inversely related to the effective dielectric thickness. It is evident from the results that using aerogel film can reduce the effective dielectric thickness due to the existence of air cells. The effective dielectric constant or relative permittivity of an aerogel film is the combination of the permittivity of the air inside the pores and the solid-state material i.e. Polyimide^35^. As the permittivity of air is less than the relative permittivity of Polyimide, the effective dielectric constant reduces by excessive increase of air inside the material. Therefore, there exists a certain range for adding open-cells into the proposed aerogel film to boost the capacitive behavior of the TENG and after that, increasing the open-cell content will degrade the capacitance and so the performance of the TENG. Thus, the 50% open-cell content shows the highest performance among all cases and will be selected as the optimum aerogel film for further studies. It should be noted that even though the outputs are reduced for 60% and 70% cases, but both cases show higher outputs than the TENG with the dense film. Therefore, all aerogel samples are superior to the simple dense layer of the dielectric validating the idea of using proposed aerogel film for performance enhancement of the TENG.

**Figure 8:**
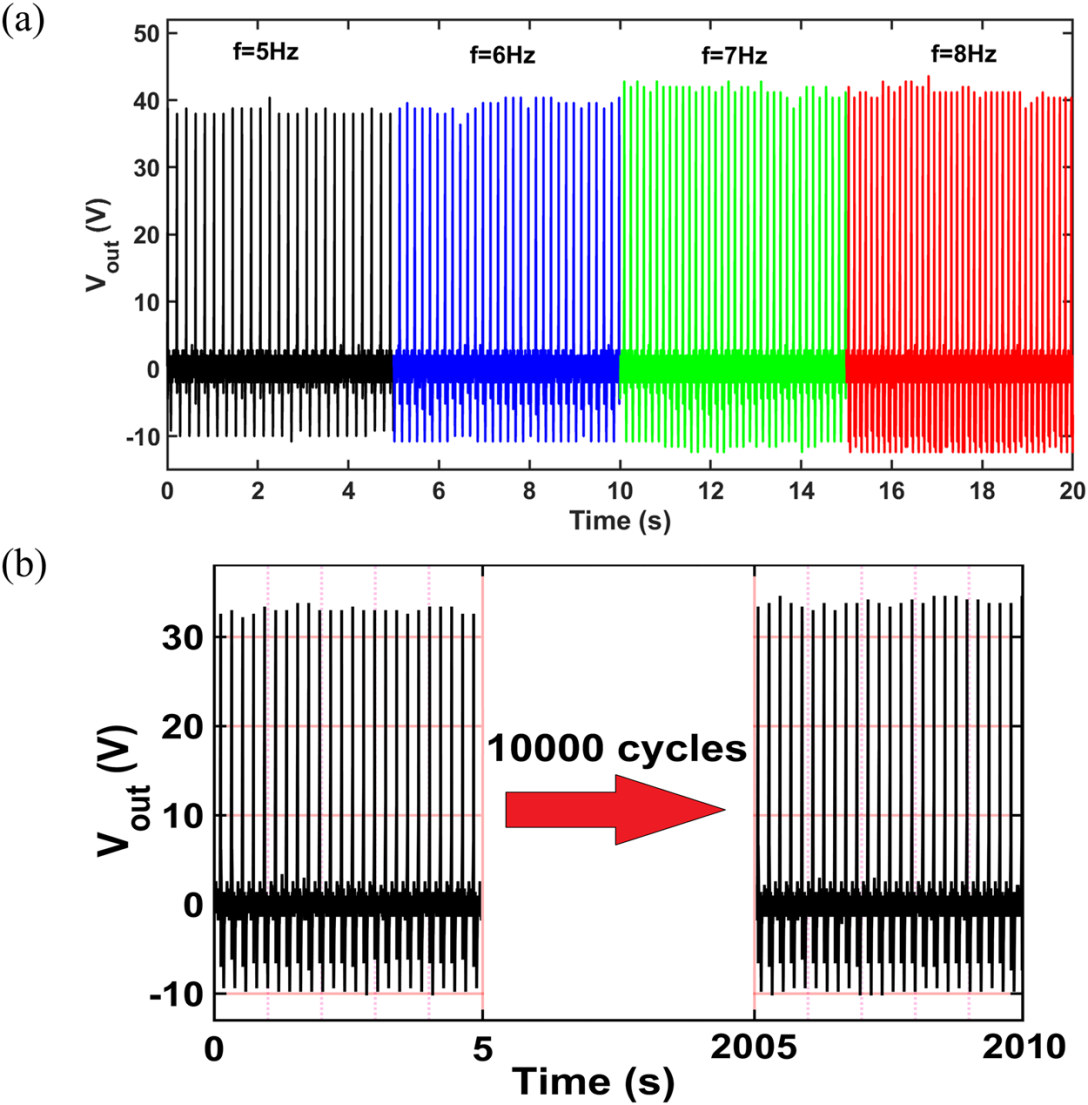
(**a**) Effect of excitation frequency on the TENG open-circuit voltage, (**b**) mechanical robustness of the aerogel-based TENG under continuous harmonic loads.

To further study the performance of the aerogel-based TENG, the effect of excitation frequency on the open-circuit voltage of the device with 50% open-cell content aerogel film has been examined, as shown in Fig. 8(a). For the lower tested frequencies, the amplitudes of voltage signals are slightly smaller than those of the higher frequencies and by increasing the frequency, the amplitudes become almost identical. The reason is from a specific value of frequency, the applied dynamic force becomes large enough such that the effective contact area will be achieved during the contact and separation. For the larger frequencies than that specific frequency, the open-circuit voltage will be independent from the frequency as the effective contact area remains constant for all frequencies^45^.

To investigate the mechanical robustness and durability of the aerogel-based TENG under harmonic loads, the TENG device with 50% open-cell content aerogel film was placed under a harmonic mechanical excitation using a shaker for 10000 cycles with 5 Hz frequency and 0.5 g acceleration. The open-circuit voltage signal was measured for this experiment, as represented in Fig. 8(b). Tt is obvious that no significant changes has been occurred on the signal which proves the robustness of the aerogel-based TENG, as also found from the compression tests in previous sections.

**Figure 9:**
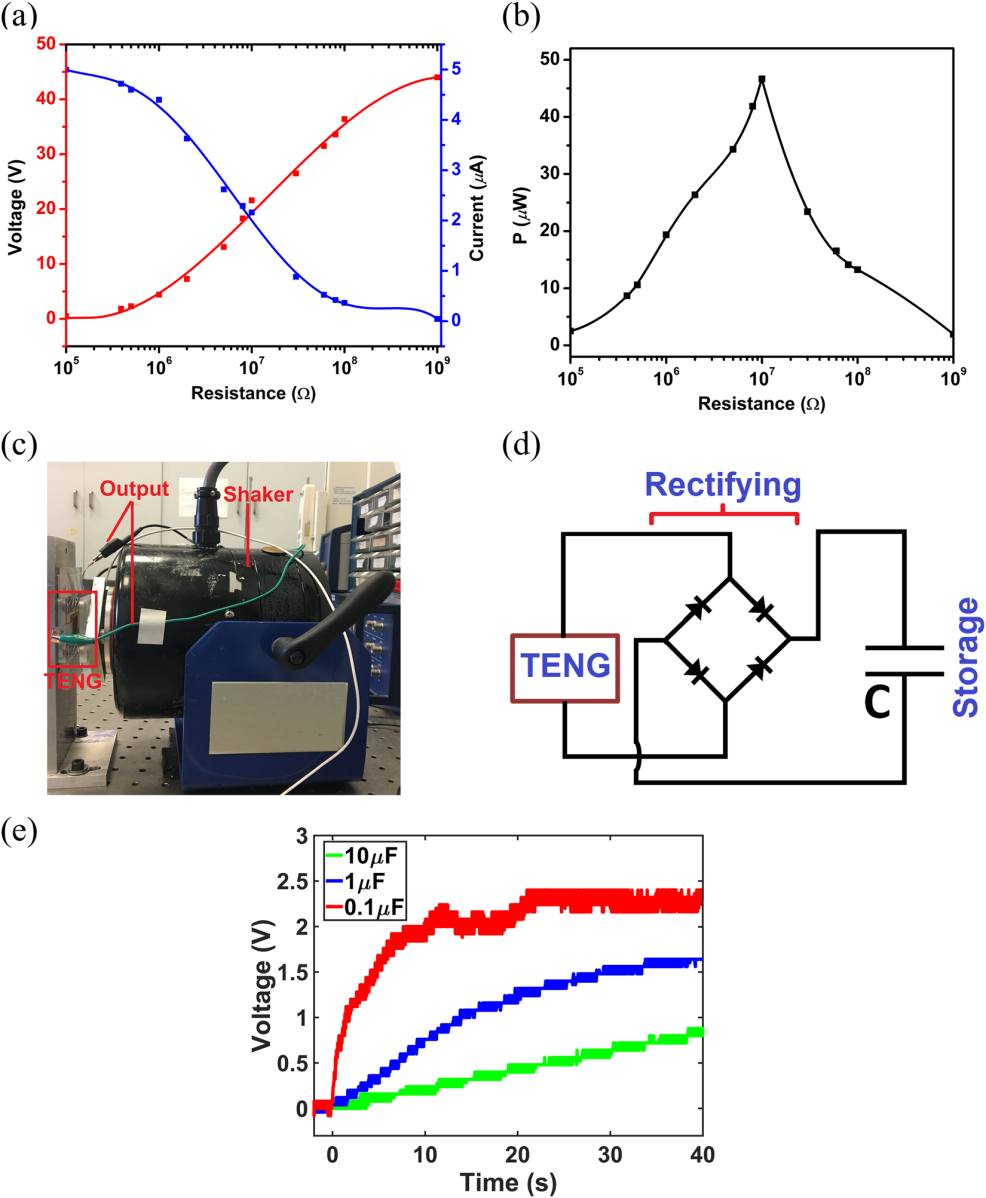
Effect of external resistive loads on: (**a**) output voltage and current, (**b**) output power. (**c**) Configuration of the dynamic test set-up, (**d**) schematics of the capacitor charging circuit by the TENG, (**e**) Voltage curves of charging different capacitors.

To practically examine the aerogel-based TENG, the device was connected to a range of external resistive loads and the electrical output characteristics were measured. Similarly, the 50% open-cell content aerogel film was used due to high performance and the frequency of 5 Hz and acceleration of 0.5 g were used in the experiments. Figure 9(a) shows the variation of output voltage and output current versus the resistance. The voltage increases by the resistance while the current decreases by the resistance due to ohmic loss. The maximum current occurs for the lowest resistance where the current curve asymptotically approaches the value of the short circuit current. Figure 9(b) shows the variation of instantaneous peak power with respect to the external resistance. The power increases by the resistance up to a maximum value and then degrades by increasing the resistance. The maximum power is around 47 *μW* which is obtained at the resistive load of $10\,M{\rm{\Omega }}$ namely optimum resistance. As it is seen, the value of optimum resistance is relatively large due to the intrinsic high internal impedance of TENG systems and this resistance can be moderated using advanced circuitry designs^46^.

To further prove the practicality of aerogel-based TENG for charging energy storage units, the device was connected to different capacitors and harmonic excitations were applied to the device at the frequency and acceleration of 5 Hz and 0.5 g, respectively. Figure 9(c) represents the testing set-up for applying harmonic loads to the TENG device where the 50% open-cell content film was used in the TENG. Figure 9(d) shows the schematic diagram of circuit for charging capacitors in which the alternating signal of the TENG is passed through a full-bridge rectifier unit to deliver DC output into the capacitor. Figure 9(e) depicts the voltage curves of charging three different capacitors including 0.1 *μF*, 1 *μF*, and 10 *μF*. It is evident that all capacitors are charged successfully by the proposed TENG. It is also observed that the maximum charging voltage and charging speed of the capacitors are increased when the capacitors become smaller, as expected.

## Conclusion

In this paper, Polyimide-based aerogel film was employed to enhance the performance of triboelectric nanogenerators. After chemically and mechanically characterization of the Polyimide aerogel, various aerogel films with different nano-porosity levels were tested. It was shown that the performance is improved when the open-cell content changes from zero to 50% and then relatively degrades for 60% and 70% open-cell contents. The enhancement is attributed to the improvement of the surface area, TENG relative capacitance, and electrostatic induction. The relative decrease on the performance of 60% and 70% open-cell contents can be attributed to the ineffective dielectric constant variation. The highest performance was achieved for 50% open-cell content where the peak open-circuit voltage, peak short-circuit current, and instantaneous power are obtained around 40*V*, 5 *μA*, and 47 *μW*, respectively. The open-circuit voltage and short-circuit current for 50% porous layer are around 8 times as much as those of a TENG with a flat and solid dielectric film. Using the 50% open-cell content film, the effect of various mechanical and electrical parameters such as frequency of excitation, cyclic loads, and external resistive loads were fully investigated. The capability of proposed TENG for charging capacitors were also demonstrated. Therefore, the proposed Polyimide-based aerogel film can significantly improve the performance of TENG systems which can be utilized for various energy harvesting and sensing applications.

## Methods

### Materials

p-phenylene diamine (PPDA) and biphenyl-tetracarboxylic acid dianhydride (BPDA) were selected as diamine and dianhydride monomers respectively. PPDA and BPDA were chosen based on their ability to from polyimide aerogels with high mechanical compression strength and modulus as high as 78.7 MPa^47^. Pyridine and acetic anhydride were used to catalyze the imidization and to scavenge water byproduct of the condensation reaction respectively^48,49^. Nmethylpyrrolidone (NMP) was selected due to its high basic aprotic nature, which can improve the imidization reaction^50,51^. 1,3,5-benzenetricarbonyl tri-chloride (BTC) crosslinking agents is chosen based on its ability to reduce the overall shrinkage along with enhancing the sample elasticity^52^. PPDA, BPDA, Pyridine, Acetic anhydride, NMP and BTC were purchased from Sigma Aldrich. All reagents were used without further purification.

### Preparation of aerogel film

PPDA-BPDA Polyimide aerogel in films geometry with about 0.5 mm thickness are prepared. To enhance the imidization process and achieve low density aerogels, diamine/dianhydride molar fraction of 25/26 and solid monomers to NMP weight fraction of 7% are selected^53^. Through preparation of polyimide sol-gels, both PPDA and BPDA monomers are added to 47% of total NMP separately and stirred for 10 min. The BTC crosslinking agent equal to 1% molar fraction of the solids is also added to remaining 6% of total NMP content and stirred until fully dissolved. Then the monomers are mixed and reacted for 5 min to form polyamic acid followed by another 3 min mixing after addition of BTC. Finally, 12.5 ml of acetic anhydride and 11 ml of Pyridine are added to the prepared solutions and stirred 3 min further. After addition of chemical reagents, the solutions are poured into the prepared 80 × 80 × 1 mm square molds and left 24 hours for the aging. After aging, the gels are removed from the mold and soaked in 100% NMP bath for 24 h to scavenge the acetic anhydride and pyridine. Then NMP content of the samples gradually exchanged with acetone in 24 h intervals to prepare them for CO_2_ super-critical drying, as CO_2_ is not soluble in NMP. The NMP to acetone exchange started with 25% acetone in NMP bath, followed by 75% acetone in NMP and finally the prepared sol-gels left in 100% acetone bath for further 72 h. 200 ml autoclave chambers are employed for the CO_2_ super-critical drying process. The process started by placing the gels into the chamber and washing them with liquid CO_2_ at 1500 Psi pressure and room temperature until complete elimination of acetone. Then the chambers are heated to 45 °C to convert the liquid CO_2_ to super critical state. Finally, the gaseous CO_2_ is vented out from the chamber at very slow rate of 10 Psi/min to avoid crack propagation through the samples. Is should be noted that Polyimide aerogel nanostructure assembly is highly sensitive to processing parameters. Therefore, varying processing parameters will result in different morphology, particle size as well as the pore size distribution. Furthermore, varying processing parameters and as in result different morphology will affect the aerogels physical, thermal and mechanical properties. For instance, varying diamine fraction in hybrid diamine polyimide aerogels has been presented as a potential approach to tailor polyimide aerogels nanostructure assembly^47,54^. Number of repeat units in polymer chain remarkably influenced surface area, shrinkage, young’s modulus, and density of the material^53,55,56^. Also, solid monomers over solvent weight fraction i.e. polymer concentration has shown a significant effect on decreasing porosity and surface area whereas increasing Young’s modulus^52,56^. In addition, other processing parameters such as imidization timing, temperature and arrangement, crosslinking agent type, crosslinking agent content as well as the ageing conditions may significantly affect polyimide aerogels morphology.

### Characterization

The Open-cell content of the aerogel foams which shows the percentage of porosities inside the material was measured by helium pycnometer (Quantachrome Instrument Ultra-Foam 1000). The Morphology of the aerogel layer was evaluated using scanning electron microscopy (Field Emission SEM, Quanta, model FEG-250). Chemical structure of the aerogel was studied based on Fourier transform infrared (FTIR) spectroscopy (Bruker ALPHA system). Thermal stability of the sample was examined by thermogravimetric analyzer (TGA) (TA-Instrument Q50) starting from room temperature up to 700 °C under nitrogen environment. Mechanical compression study was carried out to obtain Young’s modulus based on the initial slope of stress-strain curve using D695-02a ASTM guidelines (Instron 5848 micro-tester). The surface area measurement of the spongy aerogel was performed by nitrogen sorption using automated gas sorption analyzer (Autosorb iQ, Quantachrome Instruments). Finally, an electrodynamic shaker (2075E The Modal Shop Inc.) was utilized to exert harmonic excitation with controlled frequency and amplitude and the electrical outputs were measured by a digital oscilloscope (DPO 3014 Tektronix).
